# Predicting major adverse cardiovascular events during hospitalization in patients with acute myocardial infarction after percutaneous coronary intervention: Development and validation of a nomogram

**DOI:** 10.12669/pjms.41.12.13403

**Published:** 2025-12

**Authors:** Feng Zhang, Yan Yang, Defang Meng, Jun Bao

**Affiliations:** 1 Feng Zhang, Department of Cardiovascular Medicine, Affiliated Hospital of Jiangnan University, Wuxi, Jiangsu Province 21400 P.R. China; 2 Yan Yang, Department of Cardiovascular Medicine, Affiliated Hospital of Jiangnan University, Wuxi, Jiangsu Province 21400 P.R. China; 3 Defang Meng, Department of Cardiovascular Medicine, Affiliated Hospital of Jiangnan University, Wuxi, Jiangsu Province 21400 P.R. China; 4 Jun Bao, Department of Cardiovascular Medicine, Affiliated Hospital of Jiangnan University, Wuxi, Jiangsu Province 21400 P.R. China

**Keywords:** Acute myocardial infarction, Hospitalization, Major adverse cardiovascular events, Nomogram, Percutaneous coronary intervention

## Abstract

**Objectives:**

The risk of major adverse cardiovascular events (MACE) after percutaneous coronary intervention (PCI) is extremely high, and directly affects the early cardiac recovery and quality of life of patients. This study aimed to explore risk factors for MACE in patients with acute myocardial infarction (AMI) after PCI and to develop and validate a risk nomogram model.

**Methodology:**

Clinical data of 396 AMI patients who underwent PCI in the Affiliated Hospital of Jiangnan University from July 2022 to July 2024 were retrospectively selected. Patients were divided into training (n=277) and validation (n=119) cohorts based on a 7:3 ratio. The data were analyzed using the Least Absolute Shrinkage and Selection Operator (LASSO) and logistic regression, and the results were transformed into a predictive nomogram. Receiver operating characteristic (ROC) curves were drawn to evaluate the efficacy of the nomogram model and calculate the area under the curve (AUC), as well as the calibration curve and clinical decision curve (DCA).

**Results:**

The incidence of MACE was 21.2% (84/396). The identified significant predictors included no-reflow, thrombolysis in myocardial infarction (TIMI) grading, mean platelet volume and lymphocyte ratio (MPVLR), neutrophil to lymphocyte ratio (NLR), and levels of hypersensitive C-reactive protein (hs-CRP) and brain natriuretic peptide (BNP). The generated nomogram model demonstrated sufficient predictive accuracy, with AUC values of 0.961 (95% CI: 0.929-0.994) and 0.951 (95% CI: 0.894-1.000) for the training and validation cohorts, respectively. The calibration curve showed that the model’s predicted values are generally consistent with the actual values, indicating good calibration. DCA further confirmed that the predictive model has good clinical utility.

**Conclusions:**

The risk prediction nomogram model developed in this study has good predictive performance and applicability for MACE during hospitalization.

## INTRODUCTION

Acute myocardial infarction (AMI) is mainly caused by thrombus-induced acute occlusion of the coronary arteries and corresponding ischemic necrosis of myocardial cells.^1^,^2^ AMI patients are prone to shock, heart failure, sudden death, arrhythmia, etc., with a high mortality rate.^1^,^3^ The rising incidence rate of AMI among younger age groups of patients has a considerable impact on the quality of life of patients and their caregivers, and is associated with a significant socioeconomic burden.^1^,^3^,^4^

Emergency percutaneous coronary intervention (PCI) is an important clinical treatment for AMI.5 PCI can rapidly reopen occluded infarcted vessels, reduce myocardial cell injury, and restore cardiac function.^5^,^6^ However, major adverse cardiovascular events (MACE), which typically include cardiovascular death, heart failure, malignant arrhythmia, recurrent myocardial infarction, and unstable angina pectoris as defined in prior literature and clinical guidelines, may still occur after PCI surgery, impacting the prognosis of AMI patients.^6^-^8^ Therefore, early prediction of MACE risk in AMI patients after PCI is of great significance.

Currently, most research focuses on risk factors and the medium- and long-term prediction of MACE in AMI patients after PCI.^9^,^10^ However, there is limited analysis on the short-term risk factors for MACE during hospitalization. In addition, there is almost no model for predicting the risk of MACE during hospitalization in AMI patients after PCI. Nomograms are commonly used tools in the medical field, especially in oncology, to evaluate disease prognosis.^11^ This study aimed to develop a nomogram model for predicting MACE during hospitalization in AMI patients after PCI.

## METHODOLOGY

This retrospective case-control analysis examined clinical data from 396 AMI patients who underwent PCI at the Affiliated Hospital of Jiangnan University from July 2022 to July 2024. The patients were divided into a training cohort (n=277) and a validation cohort (n=119) in a 7:3 ratio.

### Ethical Approval:

The study was approved by the Ethics Review Board of the Affiliated Hospital of Jiangnan University (No. JL2024251), Date: August 25, 2025.

### Inclusion criteria:

- Patients meeting the diagnostic criteria for AMI.^1^
- Patients with indications for PCI and undergoing PCI treatment.
- First occurrence of AMI.
- The interval between onset and PCI is less than 12 hours.
- Complete clinical data.

### Exclusion criteria:

- Patients with a history of heart failure.
- Patients with old anterior myocardial infarction.
- Patients with valvular diseases and congenital heart disease.
- Patients with rheumatic heart disease and hypertrophic heart disease.
- Patients with AMI caused by coronary artery malformation and myocardial bridging.
- Patients who experience malignant arrhythmia, cardiogenic shock, and acute heart failure before PCI.
- Patients with a history of cardiac surgery.

All PCI procedures were performed by the same experienced cardiovascular interventional team within the hospital. Treatment strategies, including lesion selection, stent type, and procedural techniques, were individualized based on coronary angiography findings, yet uniformly followed the 2019 Chinese PCI Guidelines and ESC standards. This approach ensured procedural consistency and reduced potential intra-operator variability.

### Data collection:

General and clinical data were collected from all AMI patients, and included gender, age, body mass index (BMI), drinking history, smoking history, diabetes history, hypertension, cardiovascular history, heart rate, time from onset to PCI, postoperative reflow, thrombolysis in myocardial infarction (TIMI) grade, diseased vessels, diseased branches, degree of coronary stenosis, cardiac function Killip grade, serum creatinine (SCR), uric acid (UA), aspartate aminotransferase (AST), alanine aminotransferase (ALT), mean platelet volume and lymphocyte ratio (MPVLR), neutrophil to lymphocyte ratio (NLR), hypersensitive C-reactive protein (hs-CRP), white blood cell count (WBC), platelet count (PLT), albumin (ALB), hemoglobin (Hb), cardiac troponin-I (cTnI), creatine kinase isoenzyme (CK-MB), low-density lipoprotein cholesterol (LDL-C), high density lipoprotein cholesterol (HDL-C), triglycerides (TG), total cholesterol (TC), blood glucose, fragmented QRS complex (fQRS), left ventricular ejection fraction (LVEF), and brain natriuretic peptide (BNP) levels.

### Definition and Assessment of fQRS:

Fragmented QRS (fQRS) was defined according to the criteria by Das et al.^12^ as the presence of additional R`waves, notching in the R or S wave, or fragmentation of the QRS complex with a duration <120 ms, observed in at least two contiguous leads corresponding to a coronary artery territory. All 12-lead ECGs were independently assessed by two experienced cardiologists blinded to patient outcomes. In cases of disagreement, a third senior electrophysiologist made the final judgment.

### MACE definitions included:

Cardiac death, acute heart failure, malignant arrhythmia, recurrent myocardial infarction, angina pectoris, etc.

### Statistical analysis:

Data were analyzed using SPSS version 25.0 (IBM Corp, Armonk, NY, USA). The normality of the distribution of continuous variables was evaluated using the Shapiro-Wilk test. Non-normally distributed data were summarized as median and interquartile range (IQR), and the Wilcoxon test was used to compare differences between the training and validation cohorts. Normal distribution data were represented by mean ± standard deviation (SD), and an independent sample t-test was used for inter-group comparison. The count data was described by n (%), and the chi-square test was used. Cardiovascular events during hospitalization after PCI were used as the dependent variable, and the clinical data and serum indicators with differences in univariate analysis were entered into LASSO regression. This algorithm can reduce bias and multicollinearity caused by overfitting. Before modeling, all continuous variables, including BNP and hs-CRP, were evaluated for distributional characteristics. Since these variables did not exhibit substantial skewness or extreme outliers in the present sample, no normalization (e.g., z-score), categorization (e.g., quartiles), or log-transformation was applied. The original values were directly included in both LASSO regression and multivariate logistic regression analyses. This approach preserved interpretability while maintaining model stability. Ten-fold cross-validation was applied during LASSO regression to select the optimal regularization parameter (λ). The λ.min criterion was adopted, corresponding to the λ that minimizes the mean cross-validated error. All variables with non-zero coefficients selected by LASSO were subsequently included in the multivariate logistic regression model. Predictors with statistical significance (P < 0.05) were then retained to construct the final nomogram. This stepwise procedure ensured both dimensionality reduction and model interpretability. A nomogram for predicting cardiovascular events during hospitalization after PCI was developed using multiple logistic regression. The Hosmer-Lemeshow goodness-of-fit test, calibration curve, and other tests (e.g., model consistency tests) were used to evaluate the model’s fit between predicted and observed values. The C-index and the area under the receiver operating characteristic (ROC) curve (AUC) for the subjects were calculated. The discriminative power (i.e., accuracy) of the nomogram among patients was evaluated. Decision curve analysis (DCA) was used to assess the clinical value of the nomogram by calculating net benefits (NB) at different threshold probabilities. In this study, the threshold probability represents the risk of cardiovascular events during hospitalization after PCI identified by the nomogram. Test level α=0.05.

## RESULTS

### Patient characteristics:

**Fig.1:**
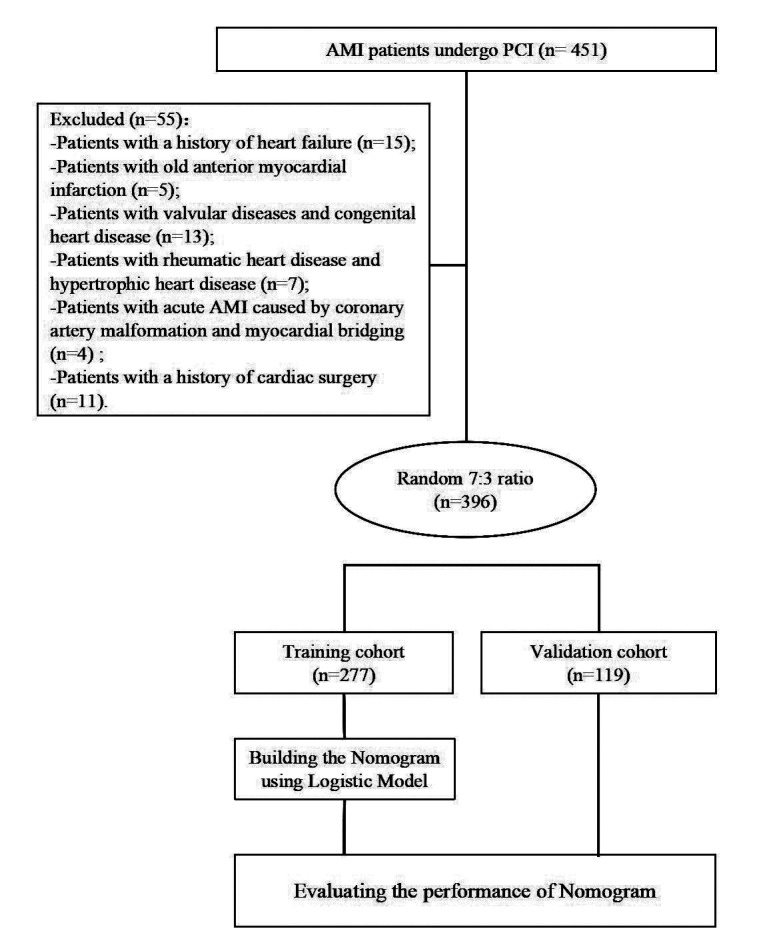
Patient inclusion process diagram; AMI: acute myocardial infarction; PCI: percutaneous coronary intervention.

This study included 396 patients with AMI who underwent PCI. The cohort included 267 males and 129 females; the age range was 51-85 years, with a median of 63 (58-67) years. A total of 84 patients developed MACE, with an incidence rate of 21.2% (84/396). Patients were randomly divided into a training cohort (n=277) and a validation cohort (n=119) in a 7:3 ratio. The screening process is shown in Fig.1. The clinical characteristics of patients are shown in Table-I. After surgery, the incidence of MACE in the training and validation cohorts was 19.9% (55/277) and 24.4% (29/119), respectively, with no statistically significant difference between the two cohorts (P=0.314). Baseline clinical characteristics of patients in both cohorts are summarized in Supplementary Table-I. Several variables, including MPVLR, NLR, Killip class, and LVEF, showed significant differences between the MACE and non-MACE groups (Supplementary Table-I), supporting their relevance for MACE prediction modeling.

**Table-I:** Comparison of clinical data between training cohort and validation cohort.

Clinical data	Training cohort (n=277)	Validation cohort (n=119)	χ^2^/Z/t	P
Male (yes), n (%)	183 (66.1)	84 (70.6)	0.775	0.379
Age (years), M (P25/P75)	63 (58-66)	64 (59-68)	-0.931	0.352
BMI (kg/m^2^), mean±SD	23.69±2.88	23.44±3.07	0.759	0.448
Drinking history (yes), n (%)	84 (30.3)	44 (37.0)	1.683	0.195
Smoking history (yes), n (%)	92 (33.2)	49 (41.2)	2.302	0.129
History of diabetes (yes), n (%)	57 (20.6)	32 (26.9)	1.904	0.168
Hypertension (yes), n (%)	74 (26.7)	29 (24.4)	0.238	0.626
Cardiovascular history (yes), n (%)	47 (17.0)	25 (21.0)	0.914	0.339
Heart rate (beats/min)	84 (76-87)	83 (75-86)	-0.968	0.333
Duration from onset to PCI (h)	5 (5-6)	5 (5-8)	-1.504	0.133
No-reflow phenomenon (yes), n (%)	263 (94.9)	109 (91.6)	1.640	0.200
***TIMI classification, n (%)***				
≤2	15 (5.4)	11 (9.2)	1.989	0.158
3	262 (94.6)	108 (90.8)		
***Pathological vessel, n (%)***				
Left anterior descending branch	144 (52.0)	54 (45.4)	1.52	0.468
Left circumflex branch	68 (24.5)	32 (26.9)		
Right coronary artery	65 (32.15)	33 (27.7)		
***Number of lesion branches, n (%)***				
Single tube	117 (42.2)	43 (36.1)	1.288	0.256
Multiple branches	160 (57.8)	76 (63.9)		
***Degree of coronary stenosis, n (%)***				
I~II	122 (44.0)	57 (47.9)	0.5	0.48
III~IV	155 (56.0)	62 (52.1)		
***Killip rating, n (%)***				
I~II	200 (72.2)	78 (65.5)	1.763	0.184
III~IV	77 (27.8)	41 (34.5)		
SCr (umol/L), M(P25/P75)	86(82-96)	85(78-96)	-1.311	0.19
UA (umol/L), M(P25/P75)	265(247-295)	269(245-295)		
AST (U/L), M(P25/P75)	45(38-53)	42(36-50)	-1.659	0.097
ALT (U/L), M(P25/P75)	48(41-56)	49(43-57)		
MPVLR, M(P25/P75)	5.35(4.58-6.03)	5.43(4.65-5.89)	-0.672	0.502
NLR, M(P25/P75)	3.56(3.22-4.55)	3.64(3.24-4.76)		
hs-CRP (mg/L), M(P25/P75)	9.45(8.58-11.44)	9.47(8.58-11.03)	-0.868	0.386
WBC (×10^9^/L), M(P25/P75)	8.57(7.76-9.49)	8.7(7.89-9.57)		
PLT (×10^9^/L), M(P25/P75)	185(165-211)	190(165-214)	-0.619	0.536
ALB (g/L), mean±SD	44.03±5.88	43.08±6.52		
Hb (g/L), M(P25/P75)	135(114-154)	138(111-158)	-0.411	0.681
cTnI (ng/ml), M(P25/P75)	0.41(0.34-0.49)	0.43(0.33-0.51)		
CK-MB (U/L), M(P25/P75)	65(62-71)	68(60-75)	-1.008	0.313
LDL-C (mmol/L), M(P25/P75)	2.55(2.16-3.01)	2.63(2.24-3.14)		
HDL-C (mmol/L), M(P25/P75)	0.85(0.66-0.98)	0.82(0.64-0.96)	-1.218	0.223
TG (mmol/L), M(P25/P75)	1.48(1.18-1.68)	1.45(1.19-1.66)		
TC (mmol/L), mean±SD	4.73±0.91	4.55±0.83	1.79	0.074
Blood glucose (mmol/L), M(P25/P75)	5.32(4.56-5.76)	5.42(4.76-5.91)		
FQRS (yes), n (%)	46 (16.6)	24 (20.2)	0.726	0.394
LVEF (%), M(P25/P75)	57(53-61)	58(52-63)		
BNP (ng/L), M(P25/P75)	157(135-184)	154(127-184)	-1.217	0.224
MACE (yes), n(%), M(P25/P75)	55 (19.9)	29 (24.4)	1.015	0.314

***Note:*** BMI: body mass index; TIMI: thrombolysis in myocardial infarction; SCR: serum creatinine; UA: uric acid; AST: aspartate aminotransferase; ALT: alanine aminotransferase; MPVLR: mean platelet volume and lymphocyte ratio; NLR: neutrophil to lymphocyte ratio; hs-CRP: hypersensitive C-reactive protein; WBC: white blood cell count; PLT: platelet count; ALB: albumin; Hb: hemoglobin; cTnI: cardiac troponin I; CK-MB: creatine kinase isoenzyme; LDL-C: low-density lipoprotein cholesterol; HDL-C: high density lipoprotein cholesterol; TG: triglycerides; TC: total cholesterol (TC); FQRS: fragmented QRS; LVEF: left ventricular ejection fraction; BNP: B-type (brain) natriuretic peptide; MACE: major adverse cardiovascular events.

**Supplementary Table-I:** Comparison of clinical data between MACE Group and Non-MACE Group.

Clinical data	MACE Group (n=84)	Non-MACE Group (n=312)	χ^2^/U/t	P
Sex (male), n (%)	51 (67.7)	216 (69.2)	1.815	0.178
Age (years), M (P25/P75)	60 (59-68)	63 (58-65)	14594	0.109
BMI (kg/m^2^), M (P25/P75)	23.30 (21.70-24.73)	23.50 (21.50-26.20)	12262	0.365
Drinking history (yes), n (%)	30 (35.7)	98 (31.4)	0.381	0.537
Smoking history (yes), n (%)	36 (42.9)	105 (33.7)	2.060	0.151
History of diabetes (yes), n (%)	26 (31.0)	63 (20.2)	3.802	0.051
Hypertension (yes), n (%)	26 (31.0)	77 (24.7)	1.047	0.306
Cardiovascular history (yes), n (%)	21 (25.0)	51 (16.3)	2.775	0.096
Heart rate (beats/min)	84.00 (74.75-90.25)	83.00 (78.00-86.00)	13490	0.679
Duration from onset to PCI (h)	5 (5-7)	5 (5-7)	13262	0.863
No-reflow phenomenon (yes), n (%)	14 (16.7)	17 (5.4)	10.040	0.002
***TIMI classification, n (%)***				
≤2	15 (17.9)	19 (6.1)	10.225	0.001
3	69 (82.1)	293 (93.9)		
***Pathological vessel, n (%)***				
Left anterior descending branch	36 (42.9)	162 (51.9)	2.550	0.280
Left circumflex branch	26 (31.0)	74 (23.7)		
Right coronary artery	22 (26.2)	76 (24.4)		
***Number of lesion branches, n (%)***				
Single tube	39 (46.4)	121 (38.8)	1.305	0.253
Multiple branches	45 (53.6)	191 (62.1)		
***Degree of coronary stenosis, n (%)***				
I~II	39 (46.4)	140 (44.9)	0.017	0.896
III~IV	45 (53.6)	172 (55.1)		
***Killip rating, n (%)***				
I~II	46 (54.8)	233 (74.7)	11.674	<0.001
III~IV	38 (45.2)	79 (25.3)		
SCr (umol/L), M(P25/P75)	93 (82-96)	85 (79-96)	14002	0.334
UA (umol/L), M(P25/P75)	265 (247-295)	265 (247-295)	13429	0.727
AST (U/L), M(P25/P75)	46 (42-51)	43 (36-52)	14604	0.107
ALT (U/L), M(P25/P75)	49 (43-56)	48 (41-56)	13213	0.907
MPVLR, M(P25/P75)	6.53 (5.46-7.85)	5.13 (4.54-5.66)	21060	<0.001
NLR, M(P25/P75)	5.31 (4.57-5.66)	3.51 (2.95-3.96)	22825	<0.001
hs-CRP (mg/L), M(P25/P75)	10.54 (9.44-13.15)	9.45 (8.55-10.51)	17717	<0.001
WBC (×10^9^/L), M(P25/P75)	8.68 (7.79-9.53)	8.57 (7.78-9.50)	13485	0.683
PLT (×10^9^/L), M(P25/P75)	194 (165-205)	185 (165-214)	13295	0.838
ALB (g/L), M(P25/P75)	43 (36-50)	45 (41-47)	11918	0.202
Hb (g/L), M(P25/P75)	137 (111-155)	136 (115-154)	12973	0.888
cTnI (ng/ml), M(P25/P75)	0.46 (0.39-0.52)	0.39 (0.32-0.48)	17026	<0.001
CK-MB (U/L), M(P25/P75)	68 (62-80)	65 (62-71)	14783	0.071
LDL-C (mmol/L), M(P25/P75)	2.62 (2.32-2.96)	2.55 (2.16-3.05)	13831	0.436
HDL-C (mmol/L), M(P25/P75)	0.86 (0.64-1.12)	0.84 (0.66-0.97)	13069	0.970
TG (mmol/L), M(P25/P75)	1.48 (1.19-1.67)	1.48 (1.19-1.68)	12317	0.399
TC (mmol/L), M(P25/P75)	4.61 (4.06-5.43)	4.59 (4.14-5.30)	13821	0.442
Blood glucose (mmol/L), M(P25/P75)	5.49 (5.13-5.84)	5.29 (4.52-5.79)	15313	0.018
FQRS (yes), n (%)	30 (35.7)	47 (15.1)	16.723	<0.001
LVEF (%), M(P25/P75)	52 (48-57)	58 (54-62)	7742	<0.001
BNP (ng/L), M(P25/P75)	170 (154-211)	154(132-174)	17292	<0.001

***Note:*** BMI: body mass index; TIMI: thrombolysis in myocardial infarction; SCR: serum creatinine; UA: uric acid; AST: aspartate aminotransferase; ALT: alanine aminotransferase; MPVLR: mean platelet volume and lymphocyte ratio; NLR: neutrophil to lymphocyte ratio; hs-CRP: hypersensitive C-reactive protein; WBC: white blood cell count; PLT: platelet count; ALB: albumin; Hb: hemoglobin; cTnI: cardiac troponin I; CK-MB: creatine kinase isoenzyme; LDL-C: low-density lipoprotein cholesterol; HDL-C: high density lipoprotein cholesterol; TG: triglycerides; TC: total cholesterol (TC); FQRS: fragmented QRS; LVEF: left ventricular ejection fraction; BNP: B-type (brain) natriuretic peptide; MACE: major adverse cardiovascular events.

### Development and validation of the nomogram model:

**Fig.2:**
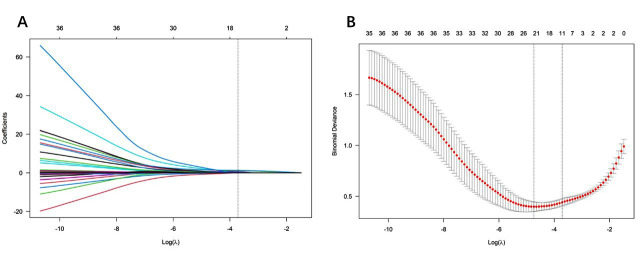
LASSO coefficient curve of MACE during hospitalization in AMI patients after PCI. A. Each curve in the graph represents the coefficient variation of each variable. The vertical axis represents the coefficient values, the lower horizontal axis represents log (λ), and the upper horizontal axis represents the number of non-zero coefficients in the model at this time. B. 10 fold cross validation fitting, then select the model.

In the training cohort, the LASSO regression algorithm was used to select influencing factors. This method helps minimize multicollinearity, providing strong predictive power and stability. Factors were selected based on the minimum partial likelihood binomial bias, and LASSO regression retained 10 non-zero coefficient variables (Fig.2). These variables were found to be significantly correlated with MACE during hospitalization after PCI. The determined variables included postoperative no-reflow, TIMI grading, Killip grading of cardiac function, MPVLR, NLR, hs-CRP, cTnI, fQRS complex, LVEF, and BNP. To further investigate the predictive significance of identified factors, a multiple logistic regression analysis was conducted using these 10 variables. The results showed that no-reflow [odds ratio (OR)=16.398; 95% confidence interval (CI)=1.577-170.549; *P*=0.019], TIMI classification (OR=12.792; 95% CI=1.675-97.673; *P*=0.014), MPVLR (OR=6.003; 95% CI= 2.899-12.431; *P*<0.001), NLR (OR=6.978; 95% CI=3.277-14.856; *P*<0.001), hs-CRP (OR=1.467; 95% CI=1.033-2.082; *P*=0.032), and BNP (OR=1.018; 95% CI=1.002-1.035; *P*=0.032) are all significant predictors of MACE in patients. The detailed results of the multiple logistic regression analysis are shown in Table-II.

**Table-II:** Analysis of adverse risk factors for MACE during hospitalization in AMI patients after PCI.

Independent variables	B	95% CI	P
No-reflow phenomenon	2.797	16.398(1.577-170.549)	0.019
TIMI classification	2.549	12.792(1.675-97.673)	0.014
MPVLR	1.792	6.003(2.899-12.431)	<0.001
NLR	1.943	6.978(3.277-14.856)	<0.001
hs_CRP	0.383	1.467(1.033-2.082)	0.032
BNP	0.018	1.018(1.002-1.035)	0.032

***Note:*** B is the regression coefficient; AMI: acute myocardial infarction; PCI: percutaneous coronary intervention; MACE: major adverse cardiovascular events; TIMI: thrombolysis in myocardial infarction; MPVLR: mean platelet volume and lymphocyte ratio; NLR: neutrophil to lymphocyte ratio; hs-CRP: hypersensitive C-reactive protein; BNP: B-type (brain) natriuretic peptide.

**Fig.3:**
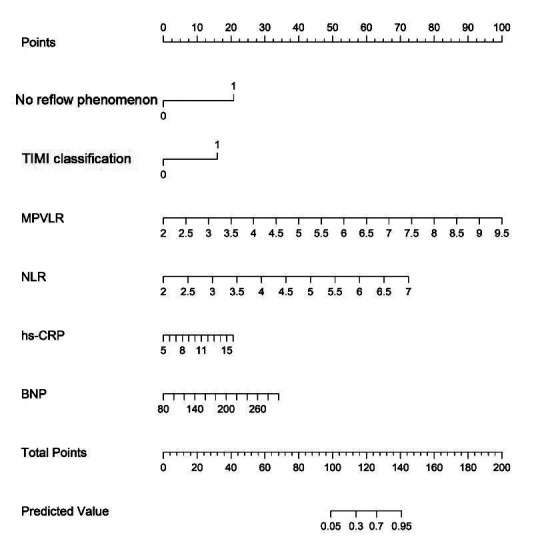
Nomogram model of MACE during hospitalization in AMI patients after PCI. Each level of the predictor variable represents a specific score. The total score is generated by summarizing the scores of each predictor variable. The total score corresponds to the MACE probability.

A nomogram model for MACE occurrence during hospitalization after PCI was then constructed based on the six independent risk factors mentioned above (Fig.3). According to the nomogram, the sum of the score values corresponding to each predictive indicator is recorded as the total score. The predicted probability corresponding to the total score represents the risk of MACE occurring during hospitalization in AMI patients after PCI.

### Application of the nomogram in a hypothetical patient

To illustrate the clinical application of the nomogram, a hypothetical patient case is presented as follows: a 68-year-old male diagnosed with AMI after PCI, with the following clinical characteristics—left ventricular ejection fraction (LVEF): 45%, neutrophil-to-lymphocyte ratio (NLR): 6.2, B-type natriuretic peptide (BNP): 720 pg/mL, high-sensitivity C-reactive protein (hs-CRP): 12.5 mg/L, and TIMI classification: 2. To illustrate how the nomogram can be applied in clinical practice, we considered a hypothetical AMI patient after PCI with the following values of the six predictors included in the final model: presence of a no-reflow phenomenon, TIMI flow grade 2, MPVLR of 7.0, NLR of 5.5, hs-CRP of 12 mg/L, and BNP of 220 pg/mL. When these values are located on the corresponding scales of the nomogram and projected upward to the “Points” axis, they correspond to approximately 40, 30, 30, 25, 20 and 15 points, respectively. The total score is therefore about 160 points, which on the “Predicted value” scale corresponds to an estimated probability of in-hospital MACE of around 70%. This case demonstrates how the nomogram can assist clinicians in identifying high-risk patients early during hospitalization. For patients with elevated predicted risk, clinicians may consider enhanced cardiac monitoring, optimization of pharmacological therapy, early initiation of rehabilitation, and individualized discharge planning to prevent major adverse cardiovascular events.

**Fig.4:**
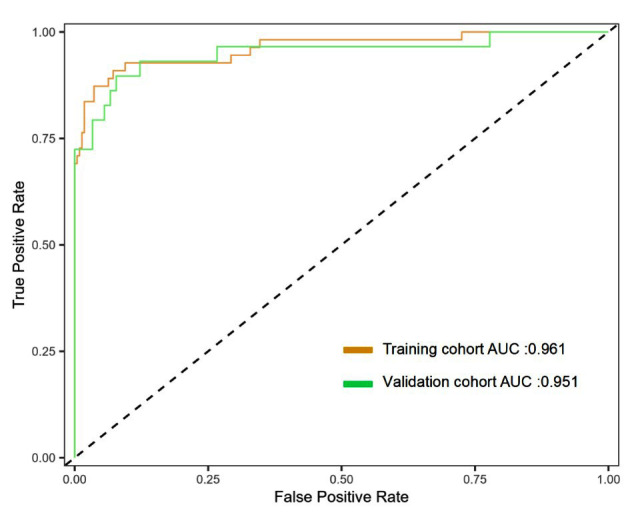
ROC curve and AUC of the nomogram model. ROC: receiver operating characteristic; AUC: under the curve.

**Fig.5:**
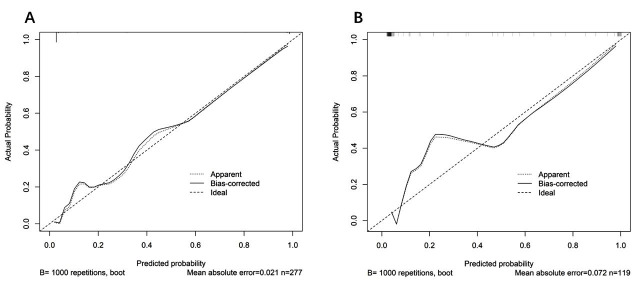
Calibration diagram of the prediction model. A. Calibration nomogram of the training cohort. B. Calibration nomogram in the internal validation cohort. The x-axis is the predicted probability of MACE during hospitalization in AMI patients after PCI. The y-axis represents the observed MACE. The diagonal dashed line represents the perfect prediction of the ideal model. The solid line represents the performance of the nomogram. The calibration curves demonstrated good agreement between the predicted and observed probabilities in both cohorts.

**Fig.6:**
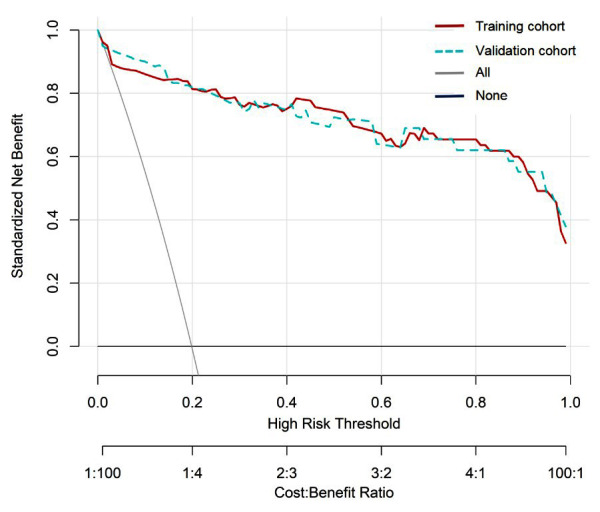
DCA of nomogram model. The x-axis displays the threshold probability, while the y-axis measures the net benefit calculated by adding true positives and subtracting false positives. DCA: decision curve analysis.

The Hosmer-Lemeshow test results were as follows: the training cohort had a χ^2^ = 11.793, P = 0.161, and the internal validation cohort had a χ^2^ = 3.509, P = 0.061. This result indicates that the predicted results are close to the observed results. The model demonstrated excellent discrimination in both the training (AUC = 0.961, 95% CI: 0.929–0.994) and validation cohorts (AUC = 0.951, 95% CI: 0.894–1.000) (Fig.4). Calibration curve analysis demonstrated a good consistency between the predicted probability and the observed MACE occurrence rate in both the training cohort and validation cohort (Fig.5). DCA curves were generated using training cohort data and validation cohort data separately (Fig.6). The DCA curves of the training cohort and validation cohort indicate that the prediction model has good clinical practicality.

## DISCUSSION

This study identified factors, such as no-reflow, TIMI grade ≤ 2, MPVLR, NLR, hs-CRP, and BNP, as risk factors for MACE during hospitalization in AMI patients after PCI. The developed nomogram model demonstrated good predictive performance and applicability.

Among the 396 AMI patients who underwent PCI in this study, 84 developed MACE during hospitalization, accounting for 21.2%. AMI is a cardiovascular disease with high mortality and disability rates.^4^–^6^ While PCI is the preferred measure for clinical treatment of AMI, and can improve patient survival rate,^6^,^13^ there is still a risk of MACE occurring after PCI.^13^ A study by Meng et al.^14^ demonstrated that 31.6% (68/215) of AMI patients after PCI developed MACE during postoperative hospitalization. The study by Ma et al.^15^ showed that the incidence of MACE during hospitalization after PCI in AMI patients was 17.3% (51/294). Rates of MACE in this study are thus consistent with previously described incidence, further emphasizing the importance of early identification of high-risk patients and the prevention of MACE to improve the prognosis and quality of life of AMI patients.

Although Killip classification is a well-established prognostic indicator in AMI patients, it was not retained in the final nomogram model. While it was initially selected by LASSO regression, it did not reach statistical significance in the subsequent multivariate logistic regression analysis. This may be attributed to moderate collinearity with other variables such as BNP and LVEF, both of which reflect cardiac function. To ensure model parsimony and avoid redundancy, Killip class was excluded from the final model. Nonetheless, Killip classification remains clinically valuable and should be considered in larger-scale validation studies.

In this study, a practical and straightforward nomogram model was developed to predict the probability of MACE occurrence during hospitalization in AMI patients after PCI. The nomogram model combines disease characteristics and objective laboratory indicators, including no-reflow phenomenon, TIMI grading, MPVLR, NLR, hs-CRP, and BNP. Most existing predictive models for MACE in AMI patients focus on mid- to long-term outcomes after PCI.^9^,^10^,^16^ This study addresses a gap by enabling the prediction of MACE in AMI patients after PCI during the initial hospitalization period. The described nomogram model may assist clinicians in identifying high-risk patients for MACE at an early stage, thereby supporting more informed and timely clinical decision-making.

The results of this study indicate that the absence of reflow is an independent risk factor for MACE during hospitalization after PCI. The study by D’Entremont et al.^17^ also showed that patients without postoperative reflow had a significantly increased risk of adverse outcomes (cardiovascular death, recurrent myocardial infarction, cardiogenic shock, or NYHA class IV heart failure) within one year. Earlier, a study by de Waha et al.^18^ also showed that the absence of reflow is closely related to hospitalization and mortality rates within one year after PCI. Therefore, for AMI patients without reflow after PCI, timely evaluation and treatment should be carried out to reduce the occurrence of MACE.

TIMI grading is a system used to evaluate coronary blood flow in patients with acute coronary syndrome (ACS). Patients with lower TIMI grades have a higher risk of developing adverse cardiovascular events.^19^ The results of this study also identified TIMI classification ≤ 2 as an independent risk factor for developing MACE during hospitalization after PCI. Bailleul et al.^19^ confirmed that TIMI grading is an independent predictor of early and late survival in ST-segment elevation myocardial infarction (STEMI) patients after PCI. This is consistent with the results of this study. However, it is important to note that the study did not differentiate AMI patients into STEMI and non-STEMI categories.

There is evidence to suggest a certain correlation between MPVLR and MACE. Specifically, higher levels of MPVLR may be associated with the occurrence and development of cardiovascular diseases such as coronary heart disease, myocardial infarction, and stroke.^20^,^21^ MPVLR can reflect the activity of platelets and the degree of inflammatory response, which are closely related to the occurrence and development of cardiovascular diseases. This study also confirmed that MPVLR is an independent risk factor for MACE during hospitalization after PCI, which is consistent with the research results of Xie et al.^21^ and Meng et al.^22^

Relevant evidence has confirmed that high NLR levels are associated with an increased risk of adverse cardiovascular events such as myocardial infarction and heart failure.^23^,^24^ A meta-analysis by Zhang et al.^23^ showed that NLR is a predictive indicator for hospitalization and long-term prognosis of STEMI patients after PCI. The study by Fagundes et al.^24^ also showed a significant correlation between NLR and the occurrence of major bleeding events and MACE. This study further confirms this conclusion. Although fQRS was retained in the LASSO regression model, it was not statistically significant in the final multivariate analysis, possibly due to collinearity with LVEF and BNP. fQRS reflects localized myocardial scarring and conduction abnormalities, and has been associated with adverse cardiac outcomes, including arrhythmia, reinfarction, and early mortality in AMI patients.^25^-^27^ Despite its non-significant result in this study, fQRS remains a clinically meaningful marker and may warrant further investigation in larger, prospective cohorts.

Kang et al. pointed out the important role of inflammatory markers, such as hs-CRP, in predicting MACE after PCI. In this study, hs-CRP was identified as an independent risk factor. As a non-specific inflammatory marker, the elevation of hs-CRP reflects the presence of an inflammatory response in the body, which can increase the risk of MACE by promoting plaque instability and thrombosis.^28^,^29^ BNP, as a sensitive biomarker for heart failure, also has important value in the prognostic evaluation of AMI patients.^30^ This study found that elevated BNP levels are an independent risk factor for MACE during hospitalization after PCI. Niccoli et al. confirmed that high levels of BNP are closely related to the incidence of MACE in AMI patients after PCI. The suggested mechanism links the elevation of BNP to impaired cardiac function and increased cardiac load in patients, thereby increasing the risk of MACE.^30^-^32^

The nomogram model developed in this study demonstrates both innovation and practical utility. The model integrates multiple risk factors, providing clinicians with an intuitive and convenient tool to assess the risk of MACE in AMI patients during hospitalization after PCI. The Bootstrap method for validation confirmed that the calibration curves of the training and validation cohorts have good overlap with the ideal curve. This indicates that the model has good calibration ability and can accurately predict the risk of patients. The Hosmer-Lemeshow verification results further supported the goodness of fit of the model. In addition to its predictive accuracy, the proposed nomogram provides actionable value for clinical decision-making. For AMI patients identified as high-risk during hospitalization, the model can support individualized interventions such as enhanced cardiac monitoring, early initiation or adjustment of cardioprotective medications (e.g., β-blockers, statins, anticoagulants), targeted patient counseling, and personalized rehabilitation planning. These measures may help reduce in-hospital complications and improve patient outcomes. Incorporating the nomogram into electronic medical record systems or clinical pathways may further facilitate its bedside use and promote risk-adapted management strategies.

The ROC curve showed that the model has high AUC, sensitivity, and specificity in predicting the risk of MACE. This provides an important basis for evaluating the diagnostic performance of the model. Furthermore, a higher AUC value indicated that the model has good discriminative ability, effectively distinguishing between patients with and without MACE. In addition, the DCA results showed that the nomogram model has a higher net benefit when implementing risk prediction. The results suggest that this model may aid clinical decision-making and improve patient management during hospitalization.

The developed prediction model has important clinical implications. Identifying risk factors for MACE shortly after the PCI procedure in AMI patients allows for the timely development of corresponding protective measures that minimize the risk of MACE in AMI patients after PCI and ensure a favorable prognosis for the disease. These measures may include:

- Close monitoring of vital signs, including heart rate, blood pressure, respiration, body temperature, etc., at regular intervals, and promptly taking corresponding measures to detect abnormal changes.
- *Psychological care*. As AMI patients may experience negative emotions such as anxiety and depression after surgery, nursing staff should actively communicate with patients, provide psychological support and comfort, and help patients build confidence in overcoming the disease.
- *Dietary care*. Patients should be guided to follow the principles of a low salt, low-fat, low sugar, and high fiber diet, avoiding overeating and controlling weight.
- *Sports rehabilitation nursing*. Personalized sports rehabilitation plans may be developed based on the specific situation of the patient. The amount of activity should be gradually increasing, from bed activities to indoor activities and then to outdoor activities, but avoiding intense exercise and overwork.
- *Medication care*. Clinicians should ensure that patients take antiplatelet aggregation, lipid-lowering, antihypertensive, and hypoglycemic medications on time and in the appropriate dosage. They should also explain the effects, side effects, and precautions of the medication to the patient.
- Health education, including popularizing relevant knowledge about AMI and PCI surgery to patients and their families, such as the causes of diseases, preventive measures, first aid methods, etc., and improving patients’ self-management abilities.
- *Sleep care:* creating a quiet and comfortable sleeping environment for patients, ensuring sufficient sleep, and promoting physical recovery.

In addition to the clinical implications, the present study offers several methodological strengths. Notably, this model is among the few predictive tools focusing specifically on in-hospital MACE after PCI in AMI patients, rather than the more commonly evaluated 30-day or long-term outcomes. The inclusion of innovative post-procedural and inflammatory indicators such as MPVLR, NLR, and BNP allows for more sensitive risk estimation tailored to the inpatient period. Moreover, the nomogram format ensures intuitive usability and enables clinicians to apply risk stratification directly at the bedside. For future research, efforts should be directed toward multicenter validation, external testing in independent cohorts, and comparative evaluation with established tools such as GRACE and TIMI scores. Additionally, integrating the model into mobile applications or AI-driven decision platforms may further enhance its clinical accessibility and real-time implementation.

### Limitations:

First, it is a single-center retrospective study with a relatively small sample size, which may introduce selection bias and affect the generalizability of the findings. Second, the model focused exclusively on the prediction of in-hospital MACE in AMI patients after PCI, without evaluating medium- and long-term outcomes. Further studies are needed to assess the prognostic value of the included variables over extended follow-up periods. Third, the study did not stratify patients by STEMI and non-STEMI subtypes, potentially limiting its applicability to specific clinical subsets. Fourth, the model was internally validated using bootstrap resampling and a hold-out validation cohort derived from the same institution. However, the absence of external validation may restrict the model’s transportability across diverse clinical populations. Future studies should aim to validate the model in multicenter cohorts or regional AMI registries. Fifth, the analysis did not adjust for perioperative medications such as dual antiplatelet therapy (DAPT), statins, or β-blockers, which are known to influence MACE occurrence. Due to incomplete documentation of drug type, dosage, and duration, medication exposure could not be reliably included in the multivariate model. Prospective studies with standardized medication data collection are warranted to better assess potential confounding effects. Finally, although this study did not perform a direct comparison between the proposed nomogram and established risk models such as GRACE or TIMI, these tools differ in their design and intended use. GRACE and TIMI scores typically predict short-term mortality or adverse events based on admission characteristics, whereas our model focuses on in-hospital risk assessment after PCI by incorporating post-procedural and inflammatory biomarkers. These differences highlight the complementary role of our model in guiding inpatient risk stratification. Future studies will include GRACE and TIMI scores as comparators for external validation and performance benchmarking.

## CONCLUSION

No-reflow phenomenon, TIMI grade ≤ 2, MPVLR, NLR, hs-CRP, and BNP are risk factors for MACE during hospitalization in patients with AMI after PCI. The nomogram model developed based on these predictors demonstrated favorable discriminative power and clinical applicability for the occurrence of MACE during hospitalization after PCI. However, due to the inherent limitations, such as a single-center design and sample size, the findings should be interpreted with caution. External validation with independent and multicenter datasets is needed before widespread clinical implementation.

### Ethics approval and consent to participate:

All procedures performed in study involving human participants were in accordance with the ethical standards of the institutional and/or national research committee(s) and with the Helsinki Declaration (as revised in 2013). The study was approved by the Ethics Review Board of the Affiliated Hospital of Jiangnan University (No: LS2024260). As this was a retrospective study and did not include any potentially identifiable patient data, informed consent was waived by the Ethics Committee of Affiliated Hospital of Jiangnan University.

### Authors’ contributions:

**FZ:** Study design, manuscript writing, revision, and validation. He also takes full responsibility for the accuracy and integrity of the data, analyses, and overall content of this study. **YY, DM and JB:** Data collection, data analysis, and critical review of the manuscript. All authors have read and approved the final version of the manuscript.
